# Covid-19 growth rate analysis: application of a low-complexity tool for understanding and comparing epidemic curves

**DOI:** 10.1590/0037-8682-0331-2020

**Published:** 2020-07-06

**Authors:** Airandes de Sousa Pinto, Edval Gomes dos Santos, Carlos Alberto Rodrigues, Paulo Cesar Mendes Nunes, Livia Almeida da Cruz, Matheus Gomes Reis Costa, Manoel Otávio da Costa Rocha

**Affiliations:** 1 Universidade Estadual de Feira de Santana, Departamento de Saúde, Feira de Santana, BA, Brasil.; 2 Universidade Estadual de Feira de Santana, Departamento de Ciências Exatas, Feira de Santana, BA, Brasil.; 3 Grupo Baiano de Oncologia, Feira de Santana, BA, Brasil.; 4 Universidade Federal de Minas Gerais, Faculdade de Medicina, Programa de Pós-Graduação em Infectologia e Medicina Tropical, Belo Horizonte, MG, Brasil.

**Keywords:** Covid-19, SARS-CoV-2, Polynomial interpolation

## Abstract

**INTRODUCTION:**

The acceleration of new cases is important for the characterization and comparison of epidemic curves. The objective of this study was to quantify the acceleration of daily confirmed cases and death curves using the polynomial interpolation method.

**METHODS:**

Covid-19 epidemic curves from Brazil, Germany, the United States, and Russia were obtained. We calculated the instantaneous acceleration of the curve using the first derivative of the representative polynomial.

**RESULTS:**

The acceleration for all curves was obtained.

**CONCLUSIONS:**

Incorporating acceleration into an analysis of the Covid-19 time series may enable a better understanding of the epidemiological situation.

The new epidemic caused by SARS-CoV-2 has revealed a pattern comprising a phase of slow growth followed by one of acceleration, a short stationary period, a peak, and, finally, a phase of deceleration (Figure 1B and Figure 2B**)**. This behavior has been observed for both new cases and deaths and varies by region, possibly due to local peculiarities, such as genetic susceptibility, climate, population density, and social inequality.

**FIGURE 1:**
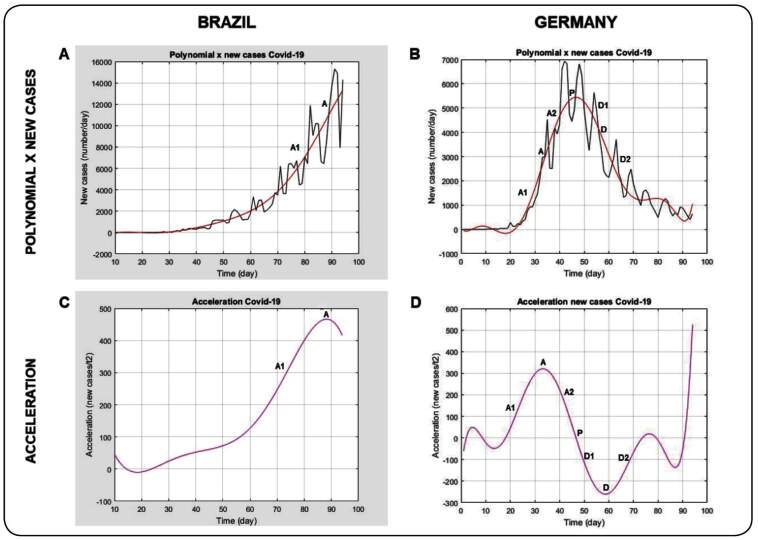
**1A:** Polynomial x curve of new cases in Brazil. **1B:** Polynomial x curves of new cases in Germany. **1C:** Instantaneous acceleration curve in Brazil. **1D:** Instantaneous acceleration curve in Germany. **A** - Maximum acceleration limit between A1 and A2 intervals. **P** - Peak of new cases, limit between the acceleration (A1 + A2) and deceleration phases of reports (D1 + D2). **D** - Maximum deceleration (absolute value), limit between D1 and D2 intervals. Intervals: **A1** - First stage of curve acceleration phase, increase in new cases. **A2** - Second stage of curve acceleration phase, increase in new cases and decrease in acceleration to zero over time. Acceleration value decreases and remains positive until reaching zero. **D1** - First stage of deceleration phase, decrease in new cases, and acceleration becomes increasingly negative until reaching D. **D2** - Second stage of deceleration phase, decrease in new cases, and acceleration returns to zero over time. New cases in Brazil = 3.8 x 10 ^-11^ day^8^ - 1.4 x 10 ^-8^ day^7^ + 1.5 x 10 ^-6^ day^6^ + 2.3 x 10 ^-5^ day^5^ - 1.4 x 10 ^-2^ day^4^ + 9.8 x 10 ^-1^ day^3^ - 2.9 x 10 day^2^ + 3.9 x 10 ^2^ day - 1.9 x 10 ^3^. New cases in Germany= 2.4 x 10 ^-9^ day ^8^ - 8.7 x 10 ^-7^ day ^7^ + 1.3 x 10 ^-4^ day^6^ - 9.2 x 10 ^-3^ day^5^ + 3.5 x 10^-1^ day^4^ - 6.5 x day^3^ + 5.4 x 10 day^2^ -1.5 x 10^2^ day + 5.6 x 10.

The diversity in the presentation of epidemic growth curves illustrates the complexity of the underlying mechanisms and the challenge of building predictive models during viral epidemics, such as Covid-19. Typically, an exponential growth model in the early phase of an epidemic is theoretically assumed. However, the literature indicates that such a premise can generate errors and overestimate the number of cases^1^ ^,^ ^2^.

The Covid-19 curve has a non-Gaussian distribution that is right- or positively-skewed, that is, there are a higher density of cases at the beginning and a lower density at the end.

Intuitively, we realized that the acceleration phase of new cases (Figure 1B and Figure 2B) is not constant; if it was, the values would have grown indefinitely and would not have generated a peak. In reality, the acceleration of COVID-19 reaches a maximum value and then decreases to zero, the point at which the Covid-19 curve reaches the peak (Figure 1D and Figure 2D). Therefore, we observe a first stage in which a concomitant increase in numbers and acceleration occurs, and a second stage, in which new cases continue to rise; however, a decrease in the acceleration occurs and reaches zero at the peak of new cases. In summary, in the first concordant phase, the new cases and acceleration increase, while in the second discordant phase, the cases increase, but the acceleration reaches zero (Figure 1B and Figure 1D, and Figure 2B and Figure 2D). The closer the acceleration value is to zero, the closer the curve will be to the peak.

**FIGURE 2:**
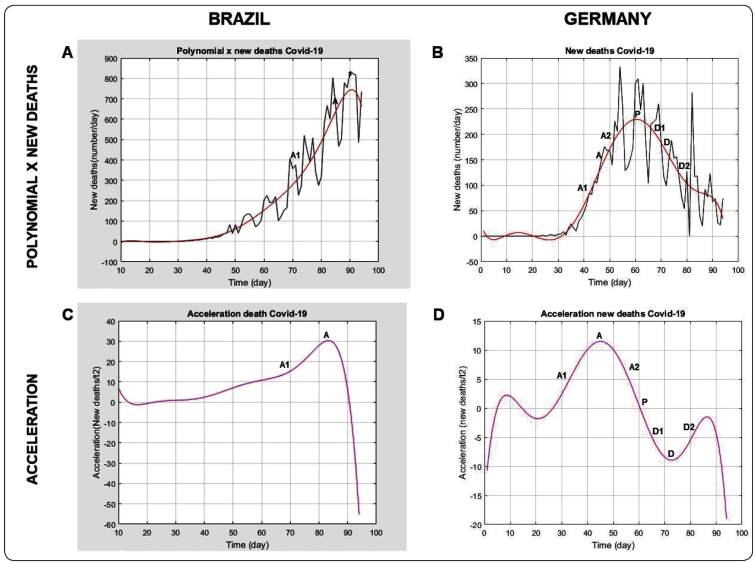
**2A:** Polynomial x daily death curve in Brazil. **2B:** Polynomial x daily death curve in Germany. **2C:** Curve of instantaneous acceleration of daily deaths in Brazil. **2D:** Curve of instantaneous acceleration of daily deaths in Germany. **A** - Maximum acceleration, limit between A1 and A2 intervals. **P** - Peak daily deaths, limit between acceleration (A1 + A2) and deceleration phases of reports (D1 + D2). **D** - Maximum deceleration (absolute value) in historical series, limit between D1 and D2 intervals. Intervals: **A1**- First stage of death curve acceleration phase, increase in deaths, and increased acceleration over time. **A2**- Second stage of curve acceleration phase, growth in death numbers, and decrease in acceleration to zero over time. Acceleration value decreases and remains positive until reaching zero. **D1**- First stage of deceleration phase, decrease in new deaths, and acceleration becomes increasingly negative until reaching D. **D2**- Second stage of deceleration phase, decrease in deaths, and acceleration returns to zero over time. Daily deaths in Brazil = -1.1 x 10 ^-10^ day ^8^ + 4.1 x 10 ^-8^.day ^7^ - 6.5 x 10 ^-6^ day ^6^ + 5.6 x 10 ^-4^ Day^5^ -2.8 x 10 ^-2^ day ^4^ + 8.5 x 10 ^-1^ Day^3^ - 1.5 x 10 day^2^ + 1.4 x 10 ^2^ day - 5.3 x 10^2^. Daily deaths in Germany = -2.6 x 10^-12^ day^8^ - 1.7 x 10^-9^ day^7^ + 7.4 x 10^-7^day^6^ - 9.9 x 10^-5^. day^5^ + 6.2 x 10 ^-3^ day^4^ - 1.9 x 10 ^-1^ day^3^ + 2.7 day^2^ - 1.6 x 10 day + 2.3.x 10.

In the deceleration phase, the acceleration begins showing a negative sign, indicating a change in the direction of the data; after the peak, the numbers begin decreasing. Like the acceleration growth phase, the deceleration phase is also not uniform; the first stage shows a decrease in numbers that is associated with an increasingly negative acceleration, and a second stage in which cases and deaths continue to decrease and the acceleration returns to zero. The second stage indicates the end of the epidemic.

The classification of phases in the epidemic curve is based on acceleration and uses only a positive or negative sign; the former indicates acceleration and the latter, deceleration. Acceleration is responsible for the slope of the curve. Recently, its value was determined by the moving regression method, which allowed a comparison of the effect of social isolation on the curves of several countries^3^.

The derivative method of differential calculus is used to find the instantaneous acceleration. However, not all curves are derivable. Currently, the epidemic curve is calculated using the 7-day moving average of discrete variables, that is, cases or deaths. The curve obtained via this method does not permit the calculation of acceleration because it is not derivable. Further, the curve is not represented by a single exponential function; the base value and exponent change at various times. It is therefore a composite function comprising several exponential functions.

In this work, we used the polynomial interpolation method to calculate the acceleration. Polynomial interpolation is an accurate, derivable, low-complexity method, and is sufficiently simple to allow its adaption to this variation of the curve, given its diverse application in several fields of expertise^4^ ^,^ ^5^. We have recently used this tool to study the pathophysiology of chagasic cardiomyopathy and mitral stenosis^6^.

This study aims to demonstrate the possiblity of calculating the instantaneous acceleration of the curves for deaths and new cases using public data from Brazil, Germany, the United States, and Russia. These countries were selected because they represent the epidemic at different stages.

The time series were extracted from the website http://www.worldometer.com/coronavirus from February 15 to May 18 (day 1 to 94), and was used for all countries studied herein. At the time of writing, February 15th was the oldest date with data available on the website and day 94 was the most recent with data available .

The peak values of the time series were obtained by reading the curves directly (Figure 1B and Figure 2B).

The MATLAB software version R2017a automatically generated a polynomial, and its degree and coefficients were adjusted to the curves of daily cases and deaths (Figure 1A, Figure 1B, Figure 2A and Figure 2B), using degree at most = 8 and Equation 1 as follows. 

Equation (1) New cases:

$$a_{n.}{day}^{n}+a_{n-1.}{day}^{n-1}{+}_{\ldots }+a_{1.}day+a_{0;}n\;\in N$$

Instantaneous acceleration was calculated on all curves using the first derivative of the polynomial (Figure 1C, Figure 1D, Figure 2C and Figure 2D). The exact time of the maximum acceleration in the ascending phase and that of the maximum deceleration in the descending phase were determined using the roots of the polynomial’s second derivative. In the downward phase, the negative acceleration was analyzed using the absolute value, and was classified as deceleration. The values of the data/days were analyzed until day 94, the end of the curve. The instantaneous acceleration was evaluated up to five days before day 89, the end date of the series, to avoid instability in the polynomial that is observed at the end of the acceleration curve.

The data for the four countries are shown in Table 1 and the curves for Brazil and Germany are shown in Figure 1 and Figure 2. These curves were chosen because they are at different stages of the epidemic. Brazil is in its initial phase whereas Germany is in the final phase and illustrates all phases of the epidemic.

**TABLE 1:** Characteristics of curves for new case and new death for the countries under study.

		Brazil		USA		Russia		Germany	
		day	number	day	number	day	number	day	number
**Daily new cases**	Maximum acceleration (new cases/day^2^)	89	466.0	40	1,690.6	75	379.0	33	320.7
	Peak (new cases/day)			50	34,517.0	87	11,656.0	42	6,933
	Maximum deceleration (new cases/day^2^)							59	-261.9
**Daily new deaths**	Maximum acceleration (new deaths/day^2^)	83	30.4	47	107.9	66	3.2	45	11.5
	Peak (new deaths/day)			67	2,683.0	92	119.0	54	333
	Maximum deceleration (new deaths/day^2^)			73	-46.1			73	-9.0

Regarding the diagnostic curve, we observed that Brazil reported its first case on the 11th day of the series and still remains in the first stage of the acceleration phase; on day 89, the new cases and acceleration still revealed an upward trend, with a value equal to 466 cases/day^2^ (Table 1, Figure 1A and Figure 1C).

Russia reported its first cases on the 20th day of the series. On day 75, a maximum acceleration occurred, equaling 379.0 new cases/day^2^. On day 87, it reached a peak of 11,656 new cases/day. This country has not yet reached its peak of maximum deceleration (Table 1). The curve is in its first stage of deceleration.

The USA reported its first new cases on day 7 of the series, and reached its maximum acceleration on day 40, at 1690.6 new cases/day², the highest value among the countries studied in our work. It peaked on the 50th day of the series, with 34,517 daily cases. The maximum deceleration has not yet been reached (Table 1) and the curve is in the first stage of the deceleration phase.

Germany reported its first cases on the 11th day, showed maximum acceleration on the 33rd day of the series with 320.7 new cases/day², and quickly peaked on the 42th, with 6,933 new cases/day. On day 59, it reached the absolute maximum value of its deceleration, with -261.0 new cases/day² (Figure 1D). After the 59th day, the number of new cases continued to decline and acceleration approached zero, thus characterizing the second stage of the deceleration phase (Figure 1B and Figure 1D**)**.

Brazil reported its first death on the 32nd day of the series and reached its maximum acceleration on the 83rd, with 30.4 new deaths/day², and is nearing the peak. The instantaneous acceleration has continued decreasing until day 89. Such behavior must be confirmed by more recent data, as it occurred in the last days of the series.

Russia had its first death on day 34 of the series, with a maximum acceleration of deaths on day 66, equal to 3.2 daily deaths/day². The curve reached the peak of daily deaths on day 92 with 119 deaths, and has not yet reached the peak of deceleration. The epidemic is in the first stage of the deceleration phase.

The USA reported its first death on day 15, and the maximum acceleration of daily deaths occurred on day 47 of the series, with 107.9 deaths/day². The acceleration continued decreasing, with a peak of daily deaths on day 67, 2,683 deaths/day. On day 73, the maximum deceleration value was reached, with -46.1 deaths/day² (Table **1**). The country is in the second phase of deceleration.

Germany’s death curve approximates resolution. The first death occurred on the 24th day of the series. The curve reached its maximum acceleration on day 45, with 11.5 deaths/day², entered the second stage of the acceleration phase and peaked on day 54, with 333 deaths/day. On day 73 of the series, the curve reached maximum deceleration, which is equal to -9.0 deaths/day², and was then followed by a decrease in daily deaths as the acceleration approached zero (Table 1, Figure 2B and Figure 2D).

This study demonstrates that the polynomial method can calculate the acceleration of epidemiological curves. The routine incorporation of this variable into an analysis of the Covid-19 time series may enable a better understanding of the curve’s phase or stage and allow the comparison of different curves, in addition to facilitating decision-making regarding epidemic containment measures.
